# Effect of the addition of a low-dose of ketamine to propofol anaesthesia on the phase-amplitude coupling features of an electroencephalogram

**DOI:** 10.1016/j.bjao.2025.100486

**Published:** 2025-09-12

**Authors:** Ryusuke Tanaka, Masahide Kaneko, Masaki Takekoshi, Satoshi Tanaka

**Affiliations:** Department of Anesthesiology and Resuscitology, Shinshu University School of Medicine, Nagano, Japan

**Keywords:** electroencephalogram, ketamine, phase-amplitude coupling analysis, propofol, total i.v. anaesthesia

## Abstract

**Background:**

Low-dose ketamine as an adjunct to propofol-based total intravenous anaesthesia (TIVA) complicates hypnotic depth monitoring by increasing bispectral index values and altering electroencephalogram (EEG). Phase-amplitude coupling is a promising EEG marker of anaesthesia-induced unconsciousness, but its response to ketamine during TIVA remains unclear. Understanding this interaction may improve hypnotic depth monitoring under multimodal anaesthesia. This study aimed to investigate the effect of low-dose ketamine on EEG modulation index and preferred phase under propofol-based total intravenous . anaesthesia.

**Methods:**

This prospective observational study analysed 19 patients (age 28–66 yr, American Society of Anesthsiology physical status 1 or 2) who underwent surgery during TIVA with propofol. After confirming the stability of propofol infusion, low-dose ketamine (0.5 mg kg^−1^) was administered as a bolus, followed by continuous infusion at a rate of 0.125 mg kg^−1^ h^−1^. Frontal EEG was analysed at baseline (PreKet) and at 10 min (PostKet1) and 20 min (PostKet2) after ketamine administration.

**Results:**

Analysis of delta-alpha phase-amplitude coupling revealed that the EEG modulation index (×10^3^) remained stable across the study period: 0.47 (95% confidence interval: 0.25–0.69) at PreKet, 0.46 (0.20–0.73) at PostKet1, and 0.35 (0.15–0.55) at PostKet2 (*P*=0.623). However, the mean preferred phase, representing the delta wave phase at which the alpha oscillation amplitude was maximal, exhibited a significant shift from 88° (95% confidence interval: 50°–126°) at PreKet to 29° (95% confidence interval: −10° to 69°) at PostKet2 (*P*=0.021).

**Conclusions:**

This study demonstrates the effect of low-dose ketamine on EEG phase-amplitude coupling during total intravenous anaesthesia with propofol. Our findings provide new insights into the neural mechanisms of low-dose ketamine and support the feasibility of phase-amplitude coupling analysis as a potential tool for improving hypnotic depth monitoring in clinical practice.

**Clinical trial registration:**

UMIN000050331.

Low-dose ketamine, a nonselective *N*-methyl-d-aspartate (NMDA) receptor antagonist, is used in general anaesthesia as part of multimodal analgesic strategies. Low-dose ketamine is defined as a continuous infusion of less than 1.2 mg kg^−1^ h^−1^ or a bolus dose of less than 1 mg kg^−1^.^1^ Because of the adverse effects and dependency risks associated with opioids,^2^ low-dose ketamine has gained attention for its opioid-sparing properties and effectiveness in perioperative pain control.^3^, ^4^, ^5^ Its use is now supported by clinical guidelines.^6^

Intraoperative administration of low-dose ketamine as an adjunct can complicate the assessment of hypnotic depth, particularly under propofol-based total intravenous anaesthesia (TIVA). Ketamine increases processed electroencephalogram (EEG) values, such as the bispectral index (BIS), when co-administered with propofol during TIVA.^7^ Its effects on EEG are dose dependent.^8^^,^^9^ EEG analysis shows that low-dose ketamine interacts with γ-aminobutyric acid (GABA)ergic anaesthetics by shifting the spectral alpha peak to higher frequencies.^10^ Additionally, low doses of s-ketamine administered during sevoflurane anaesthesia reduces the power of slow, delta, and alpha waves while increasing the peak frequency of alpha power and enhancing beta-gamma band activity.^11^ These EEG changes, along with increased BIS, may lead to misinterpretation of hypnotic depth, despite the additive hypnotic interaction between ketamine and propofol.^12^ Therefore, a new EEG analysis method for measuring hypnotic depth is needed.

Phase-amplitude coupling (PAC) in EEG refers to the modulation of higher-frequency oscillation amplitudes by the phase of lower-frequency oscillations. PAC has been investigated under various anaesthetics, with concentration-dependent PAC being observed under propofol and sevoflurane anaesthesia, which primarily act on GABA_A_ receptors.^13^^,^^14^ Thalamocortical PAC is believed to play a key role in disrupting information flow within the brain during general anaesthesia by interfering with neuronal dynamics through coupled oscillations.^15^^,^^16^ This phenomenon has been proposed as a hallmark of general anaesthesia.^15^ However, the effects of low-dose ketamine on PAC during propofol-based TIVA remain unclear.

To assess the strength of PAC, modulation index (MI) is used (Supplementary Fig. S1). A higher MI indicates stronger PAC, and an MI of zero indicates the absence of PAC.^17^ MI is lower during wakefulness and increases under surgical anaesthetic hypnotic depth.^18^ In addition to that, the preferred phase (PrP), which is defined as the phase of the delta component at which the alpha amplitude was the highest, is correlated to hypnotic depth.^13^ Therefore, this study aimed to investigate the effect of low-dose ketamine on MI and PrP under propofol-based TIVA.

## Methods

### Participants

This prospective observational study was approved by our institutional ethics review board (no. 5752) and registered in a publicly accessible database (UMIN000050331; https://center6.umin.ac.jp/cgi-open-bin/ctr/ctr_his_list.cgi?recptno=R000057312). Written informed consent was obtained from all participants. Twenty patients, aged 20–70 yr and classified as ASA physical status 1 to 2, were enrolled. All patients were scheduled to undergo elective surgery during TIVA with low-dose ketamine between March and December 2023 at the Shinshu University Hospital. The surgical procedures included abdominal, gynaecological, and breast surgeries. Patients with a history of neurological or psychiatric disorders were excluded.

### Protocol

The patients did not receive premedication before the induction of anaesthesia. All patients underwent continuous monitoring throughout anaesthesia, including non-invasive arterial blood pressure measurement, electrocardiography, pulse oximetry, end-tidal carbon dioxide (CO_2_) measurement, and BIS assessment. The BIS Quatro sensor (COVIDIEN JAPAN , Tokyo, Japan) was placed on the forehead. Epidural and peripheral nerve blocks were performed before the study based on clinical necessity. Anaesthesia was induced with propofol at an effect-site concentration of 3–5 μg ml^−1^ via a target-controlled infusion device (Terufusion, Termo, Tokyo, Japan). Anaesthesia was maintained using propofol at an effect-site concentration of 2.0–3.2 μg ml^−1^. To attenuate the haemodynamic response to tracheal intubation or surgical stimuli, a continuous infusion of remifentanil (0.07–0.33 μg kg^−1^ min^−1^) was administered and adjusted at the discretion of the attending anaesthesiologists. Rocuronium was administered to facilitate tracheal intubation or meet surgical requirements. The patients were intubated and mechanically ventilated throughout the intraoperative period. After a stable propofol infusion for at least 10 min, ketamine was administered as a 0.5 mg kg^−1^ bolus, followed by a continuous infusion at 0.125 mg kg^−1^ h^−1^.^19^ This study was conducted during the intraoperative period, beginning more than 30 min after surgery commenced. Anaesthetics were administered to maintain the BIS values between 40 and 60 before ketamine administration. After ketamine administration, adjustments to the anaesthetics were made based on the clinical judgement of the attending anaesthesiologists rather than the BIS values. Patients with modified propofol infusion rates during the study were excluded to isolate the effect of ketamine on PAC without confounding.

### Electroencephalographic recording and dataset

EEG signals were continuously recorded using a BIS Quatro sensor placed on the forehead and connected to a BIS A-3000 monitor (BIS A3000; Medtronic, Mansfield, MA, USA). Data were collected at a sampling rate of 250 Hz, with a default bandpass filter rate of 0.25–45 Hz. Electrode impedance was maintained at 5 kΩ or lower throughout the study. BIS data and spectral edge frequency 95 (SEF95) were recorded every 60 s.

Four-min EEG segments were obtained just before (PreKet), 10–14 min after (PostKet1), and 20–24 min after (PostKet2) ketamine administration. With each period, noise-free 2-min epochs were identified and subsequently analysed for comparison after the surgery was completed.

### Spectral analysis

The power spectrum and spectrogram were calculated using the multitaper spectral analysis method implemented via the pmtm function in MATLAB R2023b (MathWorks, Natick, MA, USA). The spectrum was computed with a window length of 2 minutes, no overlap, and a time-bandwidth product of three with five tapers. The group-level spectrum was obtained by averaging the data from all patients. The spectrum was presented as a spectrogram, a sequential representation of the power spectrum estimated using consecutive EEG data windows. Power was expressed as 10 times the logarithm base 10 of the squared amplitude.

### Phase-amplitude coupling analysis

Details of PAC analysis are provided in Supplementary Figure S1. The PAC between the delta (1–4 Hz) phase and the amplitudes of the alpha (8–14 Hz) bands was examined. The MI was defined as an adaptation of the Kullback–Leibler (KL) distance, a measure used to quantify the distance between two distributions. This index indicates the degree to which an empirical amplitude-distribution-like function over phase bins deviates from a uniform distribution.^17^

Using the Hilbert transform, the time series of the phases of the delta-frequency component (denoted as ϕ _*f*d_ [t]) and the amplitude of the alpha-frequency component (denoted as A_*f*a_ [t]) were obtained. The amplitude of the alpha-frequency (*f*a) band for each phase of the delta-frequency components was represented by the composite time series (ϕ_*f*d_[t], A_*f*a_[t]). After binning the phases ϕ_*f*d_ into 18 bins, the average A_*f*a_ for each phase bin was calculated. For each phase bin j, the mean amplitude was denoted by ⟨A_*f*a_⟩(j). Finally, the mean amplitude in each phase bin was normalised by dividing each bin value by the sum of all bin values as follows:

(1) $$P\left(j\right)=\frac{<A_{fa}>\left(j\right)}{\sum_{k=1}^{N}<A_{fa}>\left(k\right)}$$

where *N* is the number of phase bins, and *P* is the relative alpha amplitude of a given phase bin. Next, the Shannon entropy of distribution *P* was calculated as follows:

(2) $$H\left(P\right)=-\sum_{j=1}^{N}P\left(j\right)\log\left[P\left(j\right)\right]\text{.}$$

The MI was then determined using the KL distance to assess the PAC, which quantifies the difference between distribution *P* and uniform distribution *U*. The KL distance was derived from the Shannon entropy of distribution *P*, and the MI was calculated by dividing the KL distance by log (*N*) as follows:

(3) KL(P,U)=log(N)−H(P)

(4) $$\text{MI}=\frac{\text{KL}\;\left(P,U\right)}{\log\left(N\right)}$$

A modulogram was generated by averaging the relative alpha amplitudes over time and across delta phase bins for all subjects. Additionally, the PrP was calculated, defined as the phase of the delta component at which the relative alpha amplitude was the highest.

### Statistical analysis

The primary outcome of this study was the change in MI after ketamine administration. However, as no previous studies have established significant changes in MI, statistical methods were not used to determine the sample size. Instead, the sample size was based on previous experience with a similar study and aligned with commonly used sample sizes in this field.^20^

Continuous variables are expressed as mean (95% confidence interval [CI]), unless otherwise specified. The normality of data distribution was assessed using the Shapiro–Wilk test. The differences in BIS and EEG parameters across different time points (PreKet, PostKet1, and PostKet2) were evaluated using repeated-measures analysis of variance, followed by the Sidak test for *post hoc* comparisons. If normality was not confirmed, the Friedman test was applied, with the Wilcoxon signed-rank test for *post hoc* analysis. The Rayleigh test was conducted to analyse the PrP distribution. If the PrP distribution was found to be non-uniform, the Watson–Williams test was used to compare the mean direction of the PrP between time points. Statistical analyses were performed using SPSS (v27, IBM Corporation, Armonk, NY, USA) and the Circular Statistics Toolbox in MATLAB R2023b. Statistical significance was defined as a two-sided *P*-value of <0.05.

## Results

### Patient characteristics

**Fig 1:**
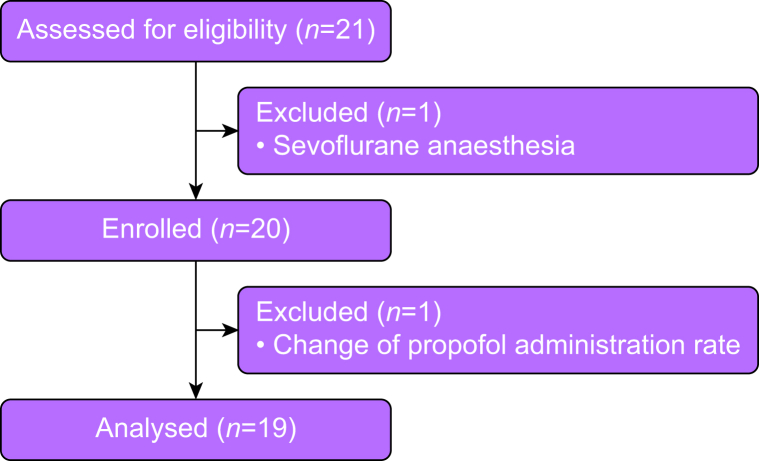
Patient flow chart. A flow diagram illustrating patient screening, enrolment, and reasons for exclusion from the study.

**Fig 2:**
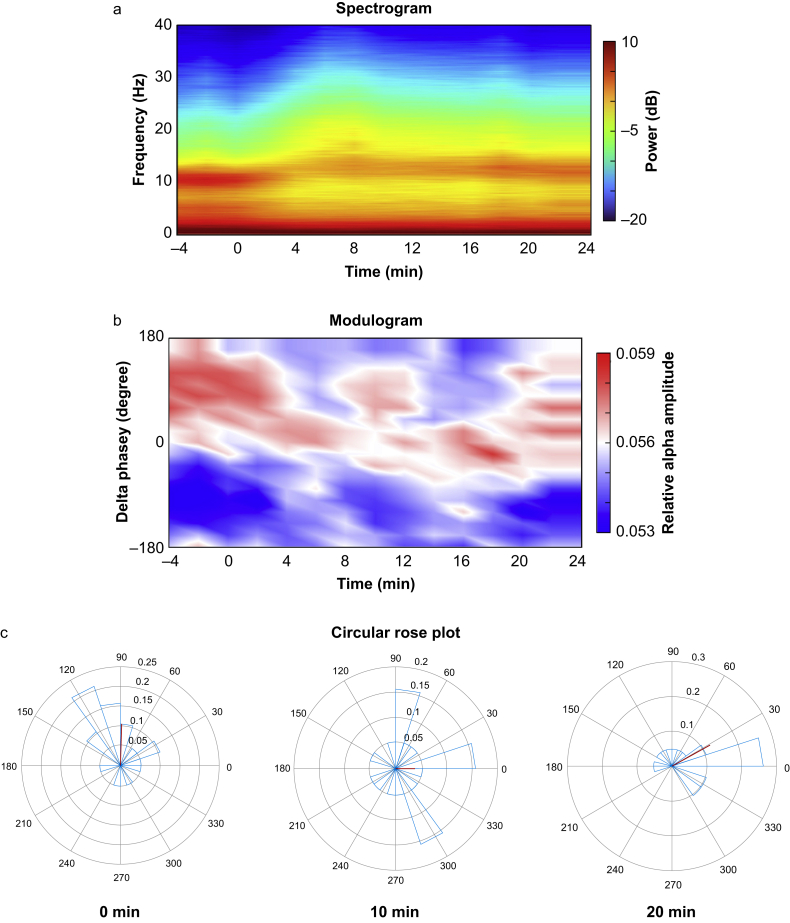
Effects of low-dose ketamine on neural oscillations over time. (a) Group-level mean of a spectrogram depicting the temporal evolution of frequency power (decibels) over time (minutes). (b) Group-level mean of a modulogram illustrating the phase-amplitude coupling of neural oscillations, with delta phase (degrees) plotted against the relative alpha amplitude over time. (c) Circular rose plot displaying the delta-band phases at which the alpha power is strongest for individual patients, represented as an angular histogram at different time points (0, 10, and 20 min). The red line indicates the mean alpha amplitude vector across all patients.

A total of 20 female patients who underwent elective surgery were enrolled (Fig. 1). The characteristics of patients are shown in Table 1. All enrolled patients were female owing to our institutional practice of preferring TIVA in young women to reduce the risk of postoperative nausea and vomiting. One patient was excluded from the analysis because of a change in the continuous propofol infusion rate during the study period, as determined by the attending anaesthesiologist. Therefore, only 19 patients were included in the final analysis. The patient’s vital signs remained within normal ranges throughout the study. Mean (sd) blood pressure was 81 (11) mm Hg at PreKet, 83 (11) mm Hg at PostKet1, and 83 (10) mm Hg at PostKet2 (*P*=0.272). Heart rate was 62 (10) beats min^−1^ at PreKet, 63 (7) beats min^−1^ at PostKet1, and 65 (12) beats min^−1^ at PostKet2 (*P*=0.234). As depicted in Figure 2, a group-level visualisation of the spectrograms is presented. The spectrogram (Fig. 2a) showed a shift in the peak alpha power frequency towards higher values, along with a decrease in alpha power after ketamine administration. Additionally, the modulogram represents that delta phase where relative alpha amplitude is high shifted from 180° (trough side) towards 0° (peak side) after low-dose ketamine administration (Fig. 2b).

**Table 1:** Patient characteristics (*n*=20). Data are expressed as median (25th–75th percentile) or number.

Sex female	20
Age (yr)	54 (50–61)
Weight (kg)	54 (51–60)
Height (cm)	159 (156–163)
Type of surgery	
Gynaecological	12
Breast	7
Abdominal	1

### Bispectral index and spectral analysis

The BIS index and SEF95 significantly increased at PostKet1 and PostKet2. The BIS values were 64 (95% CI: 60–69) and 62 (95% CI: 58–66) at PostKet1 and PostKet2, respectively, compared with 46 (95% CI: 42–51) at PreKet (*P*<0.001 for both). Similarly, SEF95 increased to 21 Hz (95% CI: 20–23) and 20 Hz (95% CI: 19–22) at PostKet1 and PostKet2, respectively, compared with 15 Hz (95% CI: 14–16) at PreKet (*P*<0.001 for both). Spectral analysis revealed significant changes in power across frequency bands after ketamine administration. Compared with the PreKet condition, beta power increased significantly at both PostKet1 and PostKet2 (−3.0 [95% CI: −4.7 to −1.4] dB and −3.8 [95% CI: −5.4 to −2.1] dB *vs* −6.6 [95% CI: −7.8 to −5.4] dB, *P*<0.001 and *P*<0.001, respectively). By contrast, the alpha, theta, and delta powers significantly decreased at PostKet1 and PostKet2. The alpha power was lower than the value at PreKet (2.9 [95% CI: 0.9–4.9] dB and 3.8 [95% CI: 1.8–5.9] dB *vs* 5.7 [95% CI: 3.9–7.6] dB, *P*<0.001 and *P*<0.001, respectively). Similarly, the theta power decreased (1.1 [95% CI: −0.4 to 2.6] dB and 1.3 [95% CI: −0.2 to 2.8] dB *vs* 3.3 [95% CI: 2.1–4.6] dB, *P*<0.001 and *P*<0.001, respectively). The delta power also exhibited a significant reduction (8.1 [95% CI: 7.1–9.1] dB and 8.0 [95% CI: 7.0–9.0] dB *vs* 9.7 [95% CI: 8.7–10.7] dB, *P*<0.001 and *P*<0.001, respectively).

### Phase-amplitude coupling analysis

No significant changes were observed in the delta-alpha PAC (MI ×10^3^: 0.47 [95% CI: 0.25–0.69], 0.46 [95% CI: 0.20–0.73], and 0.35 [95% CI: 0.15–0.55] at PreKet, PostKet1, and PostKet2, respectively; *P*=0.623) after low-dose ketamine administration. Figure 2C presents the circular rose plots of PrP. At PreKet, PrP was not uniformly distributed and was restricted to the trough side of the delta wave (mean phase: 88° [95% CI: 50°–126°], *P*=0.007). After ketamine administration, the PrP distribution became uniform at PostKet1 (*P*=0.332). However, at PostKet2, the PrP was again restricted to the peak side of the delta wave (mean phase: 29° [95% CI: −10° to 69°], *P*=0.010). Additionally, the mean direction of PrP at PostKet2 differed significantly from that at PreKet (*P*=0.021).

## Discussion

This prospective, observational, single-centre study demonstrated that low-dose ketamine increased the BIS values and SEF95 during TIVA with propofol. Spectral EEG analysis revealed a decrease in alpha and delta power and an increase in beta power after low-dose ketamine administration. These findings were consistent with those of a previous study.^11^ Under propofol-based anaesthesia, the EEG is typically characterised by slow delta and alpha oscillations. A reduction in alpha power could be the result of a decrease in the effect-site concentration of propofol.^21^ However, these spectral changes and the observed increase in BIS do not necessarily indicate a reduction in anaesthetic depth after ketamine administration. Ketamine and propofol exhibit an additive interaction in achieving hypnotic endpoints, such as unresponsiveness to verbal commands and loss of eyelash reflex.^12^^,^^22^ Therefore, when low-dose ketamine is used intraoperatively, these EEG changes should be interpreted with caution to avoid misjudging the depth of anaesthesia.

In contrast, the PAC analysis revealed a different aspect of the hypnotic state. Despite the administration of low-dose ketamine, MI remained stable, whereas the PrP shifted from approximately 90° to 30°. This shift reflects a transition from a trough-max to a peak-max pattern, which has been associated with profound unconsciousness.^13^ These findings are consistent with a previous study showing that PAC between delta and higher-frequency oscillations remained preserved after the addition of low-dose ketamine during sevoflurane anaesthesia, with the delta phase driver shifting from π to π/4.^14^ These results suggest that PAC analysis provides insights into anaesthetic hypnotic depth beyond conventional spectral methods. During TIVA with propofol, the observed shift toward a peak-max pattern after ketamine administration may indicate a deepening of the hypnotic state, supporting the utility of PAC metrics in monitoring anaesthetic hypnotic depth.

This study demonstrates the effect of low-dose ketamine on EEG PAC during TIVA with propofol. A key strength of this study is that the analysed data were obtained during an actual surgical procedure under general anaesthesia with multimodal analgesic techniques, demonstrating the feasibility of PAC analysis in a clinical setting. However, certain limitations must be considered when implementing PAC analysis in the operating room. Cross-frequency coupling is sensitive to factors such as frequency band selection, noise, and sharp signal transitions.^23^ More importantly, a major drawback of cross-frequency analysis is its limited time resolution.^23^ As in previous studies, PAC MI was calculated using 2-min EEG epochs, which may hinder the real-time assessment of anaesthetic depth—a crucial factor in intraoperative monitoring. Nevertheless, a recently developed method utilising the state-space phase-amplitude modulation algorithm has the potential to overcome this limitation.^23^ Our findings, therefore, support the feasibility of applying PAC analysis in multimodal analgesia-based clinical practice and highlight the need for the advancement of a PAC-based monitoring system.

In this study, the mean PrP of the delta-alpha PAC shifted from approximately 90° to 30°. This shift suggests a transition from a trough-max pattern—typically associated with a light hypnotic state—to a peak-max pattern, which is indicative of a deeper hypnotic state.^16^ These findings provide novel insights into the neural circuit mechanisms underlying the effects of low-dose ketamine during TIVA with propofol. Previous research suggests that the thalamus serves as the primary source of propofol-induced PAC, with the degree of hyperpolarisation in the thalamus being a key factor in determining whether PAC exhibits a trough-max or a peak-max PAC pattern.^16^ Ketamine’s primary site of action within the central nervous system appears to be the thalamocortical system, where it selectively suppresses neuronal activity in specific cortical and thalamic regions while simultaneously stimulating parts of the limbic system, including the hippocampus.^24^ Given these mechanisms, we speculate that low-dose ketamine may modulate thalamic activity in a manner that drives the transition from trough-max to peak-max PAC. Further research is required to elucidate the precise mechanisms by which low-dose ketamine modulates PAC in EEG.

Our study has some limitations. First, the sample size was relatively small, determined based on a previous study that examined the BIS, spectral, and bicoherence features of EEG.^20^ However, this small sample size was sufficient to detect changes in these parameters. Consistent with the previous study, significant changes were observed in the BIS and spectral analysis. However, subtle changes in EEG PAC-MI may have been overlooked, although such minor changes are unlikely to have clinical significance. Second, data were collected during surgery; therefore, the influence of surgical noxious stimuli on PAC cannot be entirely excluded.^25^ However, we believe that the impact on our results is likely minimal, as the haemodynamic state remained stable, although these physiological parameters are not perfect indicators of nociceptive input. As Hagihira^26^ has pointed out, similar to other EEG-derived indices, PAC-based assessment of the hypnotic depth should be performed under conditions of adequate analgesia. Third, the hypnotic depth of anaesthesia before ketamine administration was adjusted based on BIS to a clinically appropriate range (40–60) because this was an observational study. However, BIS is not a perfect surrogate for EEG activity, and variability in underlying EEG activity may still exist within this range. It is possible that variations in baseline EEG activity contributed to the observed results.

### Conclusions

Our study demonstrated that although the Phase-amplitude coupling between delta and higher frequencies remained stable, the phase driver of delta oscillations shifted from the trough towards the peak after low-dose ketamine administration during propofol-based TIVA. These findings provide new insights into the neural mechanisms of low-dose ketamine and support the feasibility of Phase-amplitude coupling analysis as a potential tool for enhancing anaesthetic depth monitoring in clinical settings.

## Authors’ contributions

Study design: RT

Data collection and analysis, and drafting of manuscript: RT, MK, MT

Critical revision of the manuscript: ST

## Funding

Japan Society for Promotion of Science (grant 23K15595 to RT).

## Declarations of interest

The authors declare that they have no conflicts of interest.
