# Supplementary material for: Effect of the addition of a low-dose of ketamine to propofol anaesthesia on the phase-amplitude coupling features of an electroencephalogram
Source: BJA Open. 2025 Sep 12;16:100486. doi: 10.1016/j.bjao.2025.100486 (PMC12767687; doi:10.1016/j.bjao.2025.100486)
Supplement: Multimedia Component 1 [file mmc1.docx]

**Supplemental Figure 1. Step-by-step procedure to quantify phase amplitude coupling (PAC) of simulated electroencephalogram (EEG) by calculating Modulation Index (MI).**

The procedure begins by applying the Hilbert transform to extract the phase time series of the delta-frequency component (φ*fd*[t]) and the amplitude time series of the alpha-frequency component (A*fa*[t]) from the original EEG signal. Next, the phase values φ*fd*[t] are binned into 18 equal intervals between −180° and 180°. For each phase bin j, the corresponding amplitude values A*fa*[t] are averaged, resulting in the mean amplitude ⟨A*fa*⟩(j). These mean amplitudes are then normalized across all bins to form a probability distribution P(j), representing the relative alpha amplitude associated with each phase bin. Shannon entropy H(P) is computed to quantify the uniformity of this distribution, where higher entropy indicates a more uniform distribution of amplitude across phase bins, and lower entropy reflects concentration around specific phase bins. Entropy is highest when the distribution is uniform (no PAC), and low when it is concentrated (strong PAC). The Kullback–Leibler (KL) divergence between the observed distribution P and a uniform distribution U is then calculated as KL(P, U) = log(N) − H(P), where N is the number of bins. Finally, the Modulation Index (MI) is obtained by normalizing the KL divergence by log(N), yielding a value between 0 and 1 that quantifies the strength of PAC. Modulogram is a time-resolved representation of the relative alpha amplitude over the delta phase using consecutive windows of EEG. MI quantifies how strongly the amplitude of alpha waves is modulated by the phase of delta waves, but it does not indicate the specific delta phase at which the alpha amplitude reaches its maximum. As shown, a peak-max pattern and a trough-max pattern can yield the same MI value. In contrast, a modulogram allows us to visually identify the phase at which the alpha amplitude reaches its maximum.
